# Case Report: Dexmedetomidine for Intractable Clusters of Myoclonic Jerks and Paroxysmal Sympathetic Hyperactivity in Progressive Encephalomyelitis With Rigidity and Myoclonus

**DOI:** 10.3389/fneur.2021.703050

**Published:** 2021-07-12

**Authors:** Yuzo Fujino, Kensuke Shiga, Masatoshi Hori, Aiko Tamura, Takahiro Iizuka

**Affiliations:** ^1^Department of Neurology, Matsushita Memorial Hospital, Moriguchi, Japan; ^2^Department of Neurology, Kyoto Prefectural University of Medicine Graduate School of Medical Science, Kyoto, Japan; ^3^Department of Emergency Medicine, Matsushita Memorial Hospital, Moriguchi, Japan; ^4^Department of Neurology, Kitasato University School of Medicine, Sagamihara, Japan

**Keywords:** α2-adrenergic receptor, dexmedetomidine, glycine receptor, myoclonus, paroxysmal sympathetic hyperactivity, stiff–person syndrome

## Abstract

**Introduction:** Progressive encephalomyelitis with rigidity and myoclonus (PERM) is a severe form of stiff-person spectrum disorder characterized by painful spasms, myoclonic jerks, hyperekplexia, brainstem dysfunction, and dysautonomia, which is sometimes resistant to γ-amino-butyric acid (GABA)-ergic agents. The response to immunotherapy varies depending on identified autoantibodies. We report a dramatic response to dexmedetomidine in a patient with glycine receptor (GlyR) antibody-positive PERM who developed intractable clusters of myoclonic jerks and paroxysmal sympathetic hyperactivity (PSH) that was highly refractory to conventional symptomatic treatment with GABAergic drugs and immunotherapy.

**Case Presentation:** A 62-year-old Japanese man was transferred to our center for intermittent painful spasms that progressed in severity over the preceding 7 weeks. On admission, he had gaze-evoked nystagmus, and paroxysmal painful spasms/myoclonic jerks triggered by sound or touch. The myoclonic jerks rapidly worsened, along with the development of hyperekplexia, opisthotonus, and PSH, leading to prolonged apnea requiring mechanical ventilation. Brain and spinal cord magnetic resonance imaging was unremarkable. Cerebrospinal fluid (CSF) examination revealed mild pleocytosis and oligoclonal bands. Surface electromyography confirmed simultaneous agonist-antagonist continuous motor unit activity. Based on the clinico-electrophysiological features, PERM was suspected. He was initially treated with intravenous steroids, immunoglobulin, benzodiazepines, and propofol, but the symptoms persisted. On day 9, he received a continuous infusion of dexmedetomidine, which resulted in dramatic reduction in the frequency of clusters of myoclonic jerks and PSH. The effect of dexmedetomidine was confirmed by surface electromyography. The addition of plasma exchange resulted in further clinical improvement. GlyR antibodies were identified in the CSF but not the serum, leading to the diagnosis of GlyR antibody-positive PERM.

**Conclusions:** PERM is an immune-mediated disorder, but dexmedetomidine, a highly selective α2-adrenergic agonist, may alleviate paroxysmal symptoms by decreasing noradrenergic neuronal activity, resulting in attenuation of antibody-mediated disinhibited increased motor and sympathetic activity. Dexmedetomidine may be useful as an adjunctive symptomatic therapy in PERM and related disorders.

## Introduction

Progressive encephalomyelitis with rigidity and myoclonus (PERM) is a severe form of stiff-person spectrum disorder (SPSD) characterized by marked rigidity, painful spasms, myoclonic jerks, hyperekplexia, brainstem involvement, long-tract signs, and prominent dysautonomia (1–3). In SPSD, several autoantibodies against either the neuronal surface or intracellular proteins have been identified; among those, glycine receptor (GlyR) antibodies are identified in ~20% of patients with SPSD, especially those with a PERM phenotype (4), and they are currently considered pathogenic antibodies. The GlyR antibodies have been shown to disrupt inhibitory glycinergic neurotransmission in the brainstem and spinal cord through internalization of the receptor, causing disinhibition of the alpha motoneurons, leading to spontaneous or reflex myoclonus and hyperekplexia, as well as sustained muscle contraction that manifests as muscle stiffness and rigidity (1–3, 5, 6). In general, pharmacologic agents that enhance γ-amino-butyric acid (GABA)-ergic neurotransmission improve these symptoms in patients with SPSD or PERM (7, 8).

Herein, we report a dramatic response to dexmedetomidine, a highly selective α2-adrenergic receptor agonist, in a patient with PERM who developed intractable clusters of myoclonic jerks and paroxysmal sympathetic hyperactivity (PSH) that was highly refractory to conventional symptomatic treatment with GABAergic drugs and first-line immunotherapy. We also discuss the potential pharmacological mechanism of action of dexmedetomidine in this disorder.

## Case Description

A 62-year-old Japanese man was transferred to Matsushita Memorial Hospital in January 2019 with progressive intermittent painful spasms in his legs. The patient had been in his usual state of health until 7 weeks before his transfer, when painful spasms developed in his right calf muscle several times a day, with each episode lasting a few seconds. The symptoms gradually worsened, with increased frequency and duration of episodes and spreading of the spasms to the contralateral side. Painful spasms were even provoked by a light touch to his legs, and he ultimately became unable to walk. Six days before his transfer to our hospital, the patient was taken by ambulance to the emergency room of another hospital. After admission there, he was treated with intravenous diazepam and oral clonazepam, but bouts of spasms developed that ascended to his truncal muscles. Three days before transfer to our hospital, he developed diplopia. The patient was subsequently transferred to our hospital for further evaluation and treatment.

He had no prodromal viral illness, recent injury, headache, bladder or bowel dysfunction, insomnia, night sweats, or weight loss. His medical history and family history were unremarkable. He had a 56-pack-year history of smoking and drank 1 L of beer daily.

On the first day of admission, his vital signs were recorded as follows: temperature, 36.8°C; blood pressure, 118/75 mmHg; pulse, 83 beats/min; respiratory rate, 20 breaths/min; and oxygen saturation, 97% while breathing ambient air. General physical examination was unremarkable. On neurologic examination, the patient was alert, well-oriented, and cooperative; his Glasgow Coma Scale score was E4V5M6 and his cognitive function was intact. The functions of his cranial nerves were normal except for saccadic pursuit eye movements and horizontal gaze-evoked nystagmus. He had no trismus or limitation of tongue movements. Muscle tone was normal in his neck and upper limbs, but his lower limbs were spastic and stiff bilaterally. Paroxysmal painful spasms or myoclonic jerks developed approximately every 10 s in his lower trunk and both legs symmetrically, which were provoked by sudden unexpected sounds or touch to his legs, and each episode lasted a few seconds (Supplementary Video 1). He also had hyperhidrosis during the bouts of spasms, but no fasciculations or myokymia were seen. Tendon reflexes were normal in his upper limbs but hyperactive in his lower limbs, although the Babinski reflex was absent bilaterally. The remainder of the neurologic examination, including coordination and sensory examination, was normal.

His blood test results were unremarkable except for a mild anemia (Hb 11.3 g/dL) and elevated serum levels of aspartate aminotransferase (95 U/L), alanine aminotransferase (79 U/L), creatine kinase (1,412 U/L), and C reactive protein (2.57 mg/dL). Additional blood investigations were unremarkable, including angiotensin-converting enzyme; thyroid hormone; and soluble interleukin-2 receptor, ANA; antibodies to SS-A/Ro, SS-B/La, Tg, TPO, ANCA; and glutamic acid decarboxylase (GAD). A thoracic magnetic resonance imaging scan and whole-body computed tomography scan did not reveal evidence of a tumor, such as a thymoma.

**Figure 1:**
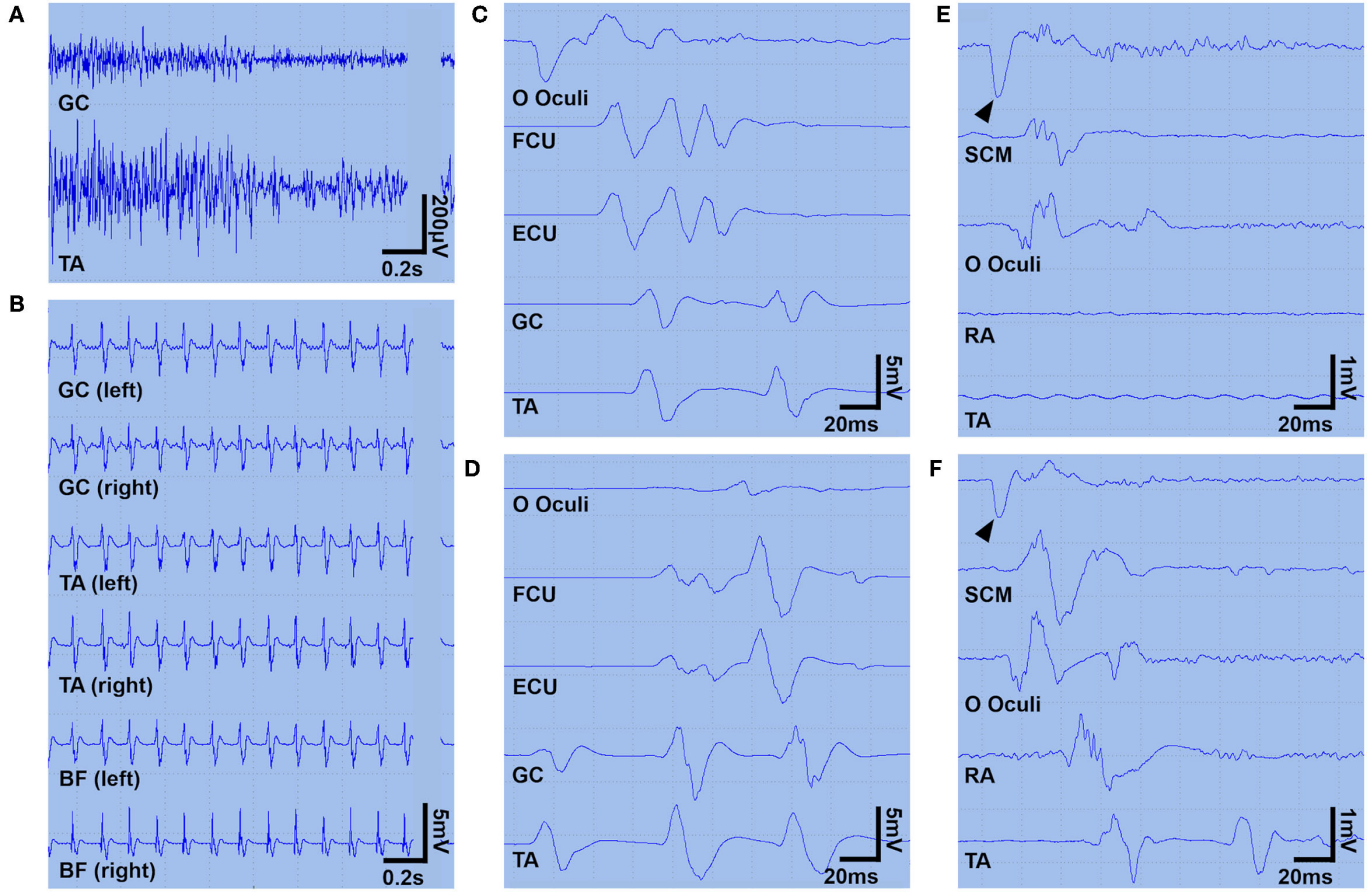
Surface EMG findings. A surface EMG **(A–D)** was recorded on day 4 under continuous infusion of midazolam (0.1 mg/kg/h) and propofol (1.0 mg/kg/h). Panel **(A)** shows the continuous simultaneous co-contraction of agonist and antagonist muscles at rest. Panel **(B)** shows ~9 Hz rhythmic bilaterally synchronous brief muscle contractions in the lower limbs (manifesting as periodic myoclonic jerks). Panels **(C,D)** show two spreading patterns descending from the facial muscles **(C)** or ascending from the leg muscles **(D)**. Myoclonic jerks were also triggered by sudden sound or touch (not shown). Another surface EMG was recorded on day 60, during a continuous infusion of dexmedetomidine (0.45 μg/kg/h) **(E)** and 90 min after discontinuation of the dexmedetomidine **(F)** to explore the inhibitory effect of dexmedetomidine on hyperexcitability of the alpha motoneurons to the jaw jerk reflex maneuver. Panel **(E)** shows that the tapping of the lower jaw (arrowheads) triggers muscle contraction limited to the SCM and OO, but panel **(F)** shows muscle contractions propagating into the TA. In panels **(A,C–F)**, a surface EMG was obtained from the left-sided muscles. BF, biceps femoris; ECU, extensor carpi ulnaris; EMG, electromyogram; FCU, flexor carpi ulnaris; GC, gastrocnemius; OO, orbicularis oculi; RA, rectus abdominis; SCM, sternocleidomastoid; TA, tibialis anterior.

After transfer to our hospital, the patient was administered a continuous infusion of midazolam and oral clonazepam; however, the paroxysmal and painful spasms/myoclonic jerks became much more severe and intractable, spreading from the lower limbs to the upper trunk, causing an opisthotonus-like posture (Supplementary Video 1). He was moved to our intensive care unit the following day. For possible tetanus and PERM, the patient was treated with human tetanus immune globulin (3,000 IU) and intravenous high-dose methylprednisolone (IVMP, 1,000 mg/d for 5 days) from day 2, followed by oral prednisolone (50 mg/d). A brain magnetic resonance imaging and nerve conduction studies were both unremarkable. A needle electromyography (EMG) examination showed rhythmic 10 Hz discharges in the right vastus lateralis at rest, but no abnormal spontaneous discharges, including myokymic or neuromyotonic discharges, were seen. A surface EMG examination obtained on day 4 showed simultaneous agonist-antagonist continuous muscle activity (Figure 1) compatible with a diagnosis of SPSD. Bilaterally synchronized periodic myoclonic jerks in his lower limbs were also observed (Figure 1).

Despite an increased dosage of sedative drugs, the myoclonic jerks/painful spasms frequently developed as a cluster accompanied by PSH, which was characterized by sinus tachycardia (110–150 beats/min), elevated systolic blood pressure (180–240 mmHg), tachypnoea (40–50 breaths/min), hyperhidrosis, and elevated temperature. Recurrent clusters caused prolonged apnea, ultimately leading to a need for mechanical ventilatory support on day 5. Hyperekplexia triggered by sound or touch frequently developed, along with a brisk jaw jerk reflex, complete ophthalmoplegia with a lack of oculocephalic reflex, and slow tongue movements. At the peak of his symptoms, clusters of myoclonic jerks developed in the whole body lasting over 10 min accompanied by PSH. These motor and autonomic spells were ceased transiently by a bolus injection of 30 mg propofol, but recurred several times an hour despite administration of a continuous infusion of propofol (3.5 mg/kg/h at the maximum dose), midazolam (0.2 mg/kg/h at the maximum dose), rocuronium (0.4 mg/kg/h), and fentanyl (1.0 μg/kg/h). Cerebrospinal fluid (CSF) examination obtained on day 6 (4 days after the initiation of IVMP) revealed 26 white blood cells/μL (mononuclear cells 100%) with oligoclonal bands. The IgG index was not elevated (0.62). Polymerase chain reaction testing was negative for herpes simplex virus (HSV)1, HSV2, and varicella zoster virus. An electroencephalogram recorded on day 7 under a continuous infusion of midazolam and propofol showed frontal intermittent rhythmic delta activity. The patient also began to receive intravenous immunoglobulin (IVIg, 0.4 g/kg/d for 5 days) from day 8.

**Figure 2:**
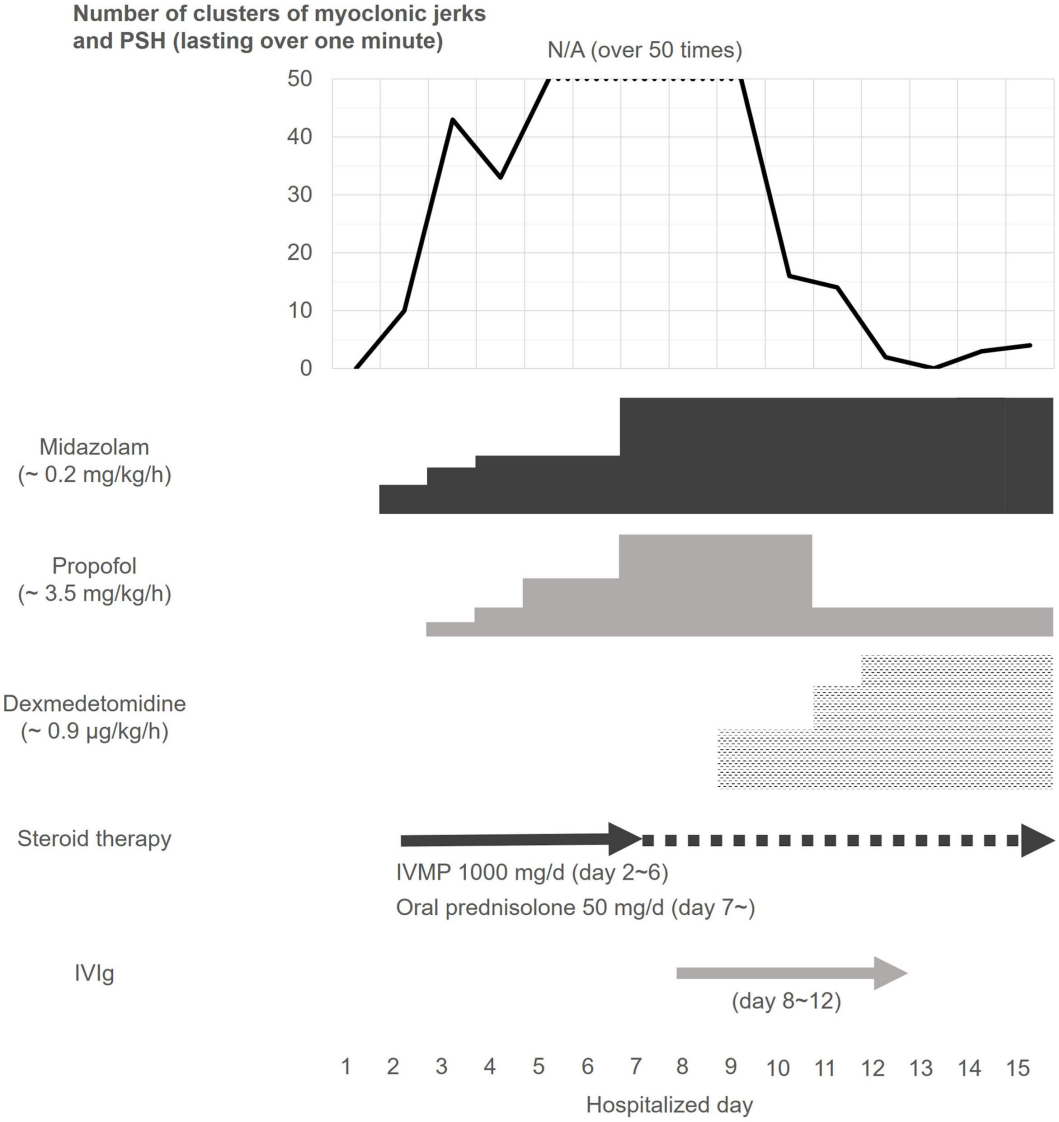
Clinical course of clusters of myoclonic jerks and paroxysmal sympathetic hyperactivity. Despite continuous infusion of midazolam and propofol, the patient frequently developed severe clusters of myoclonic jerks and PSH. The patient was also treated with a 5-day course of IVMP from day 2, and IVIg from day 8 of admission, but the clusters did not respond to the GABAergic agents and immunotherapy. However, the frequency of the clusters rapidly declined after dexmedetomidine treatment, which was started from day 9 of admission. After that, the episodic symptoms were controlled during the remainder of his hospitalization (see text). IVIg, intravenous immunoglobulin; IVMP, intravenous high-dose methylprednisolone; PSH, paroxysmal sympathetic hyperactivity.

Based on the pharmacological resistance to GABAergic drugs and immunotherapies with IVMP and IVIg, the patient was started on a continuous infusion of dexmedetomidine (a maximum dose of 0.9 μg/kg/h) on day 9. Surprisingly, after commencing dexmedetomidine, the clusters of myoclonic jerks with PSH rapidly ameliorated, without any remarkable adverse events (Figure 2). The potential inhibitory effect of dexmedetomidine on the propagation of hyperekplexia was confirmed by surface EMG (Figure 1). He was then started on a total of six cycles of plasma exchange from day 22 onward. Two days later, his symptoms improved further. His trachea was extubated, and he was able to mobilize on day 44. On day 133, he was discharged back to home. On discharge, he had hyperreflexia and mild leg stiffness but was otherwise neurologically unremarkable; his modified Rankin Scale score was 1. On discharge, he was treated with prednisolone (20 mg/d), clonazepam (4.5 mg/d), and tizanidine (3 mg/d).

## Antibody Assays

We measured the antibodies against the neuronal surface or synaptic proteins, including N-methyl-d-aspartate receptor (NMDAR), α-amino-3-hydroxy-5-methyl-4-isoxazolepropinic acid receptor (AMPAR), GABAB receptor (GABAbR), GABAA receptor (GABAaR), metabotropic glutamate receptor 5 (mGluR5), dipeptidyl peptidase-like protein 6 (DPPX), contactin-associated protein-like 2 (Caspr2), leucine-rich glioma-inactivated 1 (LGI1), and neurexin 3, as well as GlyR. These neuronal surface antibodies were measured at the laboratory of Josep Dalmau (University of Barcelona, Barcelona, Spain) using a rat brain immunohistochemistry and cell-based assay in both serum and CSF. Antibodies against classical paraneoplastic intracellular antigens (amphiphysin, CV2, GAD65, Ma2/Ta, Ri, Yo, Hu, recoverin, SOX1, titin, zic4, and Tr) were also measured in serum at Kitasato University with EUROLINE (Euroimmun AG, Lübeck, Germany).

The patient was strongly positive for GlyR in his CSF, but serum testing was negative. In addition, the patient also had extremely low levels of NMDAR antibodies in his CSF (NMDAR antibodies were not examined in the serum). Classical paraneoplastic antineuronal antibodies were negative for all 12 examined antigens.

## Discussion and Conclusions

This study shows several important findings: (1) the patient developed clusters consisting of myoclonic jerks/painful spasms and PSH refractory to continuous infusion of GABAergic agents, IVMP, and IVIg; (2) despite the lack of response to these treatments, dexmedetomidine rapidly ameliorated the symptoms; and (3) GlyR antibodies were identified in the patient's CSF.

It is important to note that the patient developed severe PSH as well as myoclonic jerks/painful spasms associated with GlyR antibodies. In this case, the myoclonic jerks/sustained spasms caused a potentially fatal apnea that required mechanical ventilatory support. PERM is generally regarded as a severe form of SPSD. One study found that 24 of 33 patients (72%) with PERM became bedridden at the peak of the disease activity, and four (12%) died (2). A variety of autonomic symptoms have also been described in patients with SPSD; paroxysmal dysautonomia may cause sudden death (9), but PSH has not been described in patients with PERM yet. PSH was originally reported in association with traumatic brain injury but has subsequently been described in other conditions, such as anoxic brain injury, stroke, brain tumors, and anti-NMDAR encephalitis (10, 11).

It is of note that PSH was seen in this case because PSH and paroxysmal motor symptoms in GlyR antibody-positive PERM may have the common pathophysiological mechanisms. The mechanism of PSH in this case remains speculative; however, as proposed in PSH, disruption of the descending inhibitory pathway from the brainstem to the spinal-reflex arcs dysregulates the balance between the excitatory and inhibitory spinal interneurons, causing excitatory spinal interneuron hyperactivity and resulting in increased motor and symptomatic output from the spine (the excitatory: inhibitory ratio model) (10, 12). Although the association between glycinergic neurotransmission and this model remains to be elucidated, glycine is an inhibitory neurotransmitter predominantly found in the brainstem and spinal cord (2, 3). Descending glycinergic neurons from the brainstem control startle reflex in the spinal cord (3). Inhibitory glycinergic neurons in the spine are also associated with reciprocal inhibition by 1a interneurons, 1b inhibition, and recurrent inhibition (3); thus, a disruption to the glycinergic neurons may increase spinal excitatory interneuron activity. Therefore, in this case, GlyR antibody-mediated disinhibition of motor and sympathetic outflow may cause clusters of PSH as well as myoclonic jerks/painful spasms.

The remarkable finding of this study is the dramatic response to dexmedetomidine, despite the patient's condition being highly resistant to the concurrent use of a continuous infusion of GABAergic agents and immunotherapies, such as IVMP and IVIg. Typically, patients with GlyR antibody-positive SPSD have a relatively favorable response to immunotherapies compared with those with antibodies against intracellular antigen (e.g., GAD) or those without antibodies (1, 4). In this case, IVMP and IVIg had already been started before the initiation of dexmedetomidine; thus, rapid reduction in the frequency of clusters may have been due to delayed effect of the immunotherapies combined with the use of dexmedetomidine, or alternatively, their synergistic effect. We cannot exclude such a possibility; however, inhibition of the propagation of hyperexcitable alpha motoneurons in the spinal cord, as shown in the surface EMG study in this case, suggests the efficacy of dexmedetomidine on GlyR antibody-mediated disinhibited hyperexcitability of the alpha motoneurons.

Dexmedetomidine is a highly potent selective agonist of the α2-adrenergic receptor, which is a major presynaptic feedback receptor. It inhibits the exocytosis of noradrenergic neurons under its stimulation (13), causing a sedative effect. Dexmedetomidine has also been shown to inhibit excitatory glutamatergic neurotransmission by decreasing the presynaptic release of glutamate (14) or promoting extracellular glutamate uptake through excitatory amino acid transporter 3 (15). Based upon the excitatory: inhibitory ratio model, dexmedetomidine might attenuate increased spinal excitatory interneuron activity, resulting in improvement of increased motor and sympathetic outflow. In addition, dexmedetomidine has been shown to have direct sympatholytic effects, since the α2-adrenergic receptors are expressed in autonomic ganglia as well as in the central autonomic system (13, 16). These pharmacological actions may have ameliorated both the motor and autonomic symptoms. Actually, dexmedetomidine was administrated as a sympathetic treatment for violent myoclonus and sympathetic storming in another PERM case (17).

In this case, GlyR antibodies were identified exclusively in the CSF, and CSF-restricted oligoclonal bands were detected. The IgG index was not elevated, but intrathecal synthesis of the antibodies was more likely. The GlyR antibodies have been reported to disrupt inhibitory glycinergic neurotransmission in cultured spinal motor neurons through direct antagonistic actions (6), or internalization of the GlyRs (2). Therefore, GlyR antibodies are more likely to be directly involved in the pathogenesis of symptoms.

The main limitation of this study is that it is a retrospective single case report. The efficacy of dexmedetomidine was examined in a prospective manner, but the concomitant use of GABAergic drugs and immunotherapy made it difficult to assess the efficacy of dexmedetomidine fully and accurately.

Despite such a limitation, we have demonstrated a dramatic response to dexmedetomidine, which may be potentially ameliorate PERM-associated symptoms *via* its unique pharmacologic action, and without affecting the immune-mediated mechanisms. Given its basic pharmacology, dexmedetomidine may also be useful as a symptomatic treatment for disinhibited sympathetic hyperactivity in patients with GlyR antibody-negative PERM, because it is effective for the management of motor symptoms and PSH associated with other conditions such as traumatic brain injury (18). Further studies are necessary to determine the efficacy of dexmedetomidine in patients with SPSD.

## Patient Perspective

I am so happy that I am able to walk again and return to my daily life. I hope that my case will be shared by medical professionals around the world to help them in their practice.

## Data Availability Statement

The original contributions presented in the study are included in the article/Supplementary Material, further inquiries can be directed to the corresponding author/s.

## Ethics Statement

Ethical review and approval was not required for the study on human participants in accordance with the local legislation and institutional requirements. The patients/participants provided their written informed consent to participate in this study. Written informed consent was obtained from the individual(s) for the publication of any potentially identifiable images or data included in this article.

## Author Contributions

YF the first author, interpreted the data, wrote the first manuscript, and is responsible for the overall content. KS, MH, AT, and TI contributed significantly to the discussion of the case and revision of the first manuscript. All authors approved the final manuscript to be published and assured all the questions regarding the accuracy of the article.

## Conflict of Interest

The authors declare that the research was conducted in the absence of any commercial or financial relationships that could be construed as a potential conflict of interest.
