# Supplementary material for: Case Report: Dexmedetomidine for Intractable Clusters of Myoclonic Jerks and Paroxysmal Sympathetic Hyperactivity in Progressive Encephalomyelitis With Rigidity and Myoclonus
Source: Front Neurol. 2021 Jul 12;12:703050. doi: 10.3389/fneur.2021.703050 (PMC8311021; doi:10.3389/fneur.2021.703050)
Supplement: Supplementary file 2 [file Data_Sheet_1.DOCX]

Supplementary Material

# Supplementary Video legends

Myoclonic jerks of the patient. On the admission day, paroxysmal myoclonic jerks developed in his legs symmetrically, which were provoked by tactile stimulation. These myoclonic jerks became more intractable, spreading from the lower limbs to the upper trunk, leading to an opisthotonus-like posture on day3.
